# Neonatal subpial hemorrhage: clinical presentation, neuroimaging findings and outcome

**DOI:** 10.1007/s00234-025-03589-y

**Published:** 2025-03-17

**Authors:** Andres Server, Anna Latysheva, Bård Nedregaard, Arild Erland Rønnestad, Pål Bache Marthinsen

**Affiliations:** 1 Section of Neuroradiology, Department of Radiology, Oslo University Hospital, Oslo, Norway; 2 Department of Neonatal Intensive Care, Oslo University Hospital, Oslo, Norway

**Keywords:** Perinatal stroke, Neonatal subpial hemorrhage, Intracranial hemorrhage, Magnetic Resonance Imaging

## Abstract

**Purpose:**

Subpial hemorrhage is a rare form of intracranial hemorrhage (ICH) in neonates that remains underreported and inadequately understood. The aim of this study is to characterize the neuroimaging patterns of subpial hemorrhage, assess changes in the underlying brain parenchyma, and examine its clinical features and outcomes.

**Methods:**

We reviewed the medical records and neuroimaging data of neonates with subpial hemorrhage admitted to our hospital between January 2010 and December 2023. Cases of subpial hemorrhages were identified through keywords searches within the hospital´s electronic database.

**Results:**

Twenty-eight patients were included in this retrospective study, 82% of whom were born at term. The most common clinical indication for imaging was a combination of apneas and seizures, ocurring in 50%. Hematologic abnormalities were present in 58% of patients. Magnetic resonance imaging (MRI) was performed acutely at the time of presentation between days 1 and 9 of life in 85% of cases. Subpial hemorrhages were unilateral in 86% of neonates, most commonly located in the temporal lobe (44%), and associated with other type of intracranial hemorrhage in 96% of cases, most often parenchymal (86%) and subdural (64%) hemorrhages. We identified three imaging patterns of subpial hemorrhage and two patterns of changes in the underlying brain parenchyma. Additionally, the hyperintense pia mater sign (HPm-sign) was observed on time-of-flight MR angiography (TOF-MRA) in 12 of 18 patients. Neurologic sequelae were noted in 28% of survivors.

**Conclusion:**

Subpial hemorrhage has a distinctive MR pattern, often accompanied with cortical infarction and in most cases underlying parenchymal hemorrhage. In this study, we identified the HPm-sign that may be used to differentiate subpial hemorrhage from other types of hemorrhages. Additionally, we found a correlation between prominent medullary veins (PMV) and intraparenchymal hemorrhage (IPH).

**Supplementary Information:**

The online version contains supplementary material available at 10.1007/s00234-025-03589-y.

## Introduction

Neonatal subpial hemorrhage is a subtype of intracranial hemorrhage that remains underrecognized and poorly understood [1, 2]. The subpial space is defined as the potential space between the glia limitans and the pia mater [1, 3, 4].

In 1972, Friede [5] described nine cases of subpial hemorrhage characterized by hematomas dissecting underneath the pia mater without evidence of hemorrhage into the subarachnoid space or into the brain parenchyma on postmortem studies. Marin-Padilla [3] in a landmark article on hemorrhagic lesions of the neocortex described cases of subpial hemorrhage including acute lesions, subacute (healing) and chronic (repaired) lesions, while exploring their neuropathology, developmental impact and outcome.

Huang and Robertson [6] reported seven term neonates with spontaneous superficial parenchymal and leptomeningeal hemorrhage on computed tomography (CT) and magnetic resonance imaging (MRI); Cain et al. [7] described 17 cases of neonates with subpial hemorrhage and found acute coagulation abnormalities to be present in 77% of cases. More recently, Assis et al. [8] documented 16 cases of subpial hemorrhage with underlying cerebral infarction, and Dabrowski et al. [2] described 31 neonates with subpial hemorrhages, 77% of whom had associated intracranial bleeds.

In this study, we aimed to characterize the MRI findings of subpial hemorrhage, with an emphasis on the appearance, changes in the underlying brain parenchyma, association with concomitant findings, comorbid medical and surgical conditions, and the size and location of subpial hemorrhage. Furthermore, we describe the risk factors, clinical presentation, and long-term outcomes of neonates with subpial hemorrhage.

## Materials and methods

### Subjects

This was a retrospective descriptive study of neonates who underwent brain MRI at Oslo University Hospital between January 2010 and December 2023. Participants were retrospectively identified by two board-certified neuroradiologists: A.S., P.B.M. with 25 and 15 years of experience in pediatric neuroradiology by using the terms “hemorrhage” and “neonate” through a key word search of our PACS database. Images were reviewed on the clinical PACS, from which 28 patients who had subpial hemorrhage were included in this study.

### Clinical information

Electronic medical records of all neonates with subpial hemorrhage identified on imaging were reviewed. A standardized data-capture form was used to collect the following data: gestational age at birth, sex, mode of delivery, APGAR score, imaging indication/clinical presentation, age at onset of symptoms, comorbid medical or surgical conditions, hematologic abnormalities, treatment, age at more recent follow-up, and outcomes.

### MRI Acquisition

MRI scans were performed using either a 1.5 T unit (Magnetom Avanto-Siemens, Erlangen, Germany) in 19 neonates or a 3 T unit (Magnetom Skyra-Siemens, Erlangen, Germany) in 9 neonates. All neonates identified underwent our standard protocol for neonatal MRI, which includes sagittal T1-weighted 3D-MPRAGE (T1WI), axial and coronal T2-weighted FSE (T2WI), and diffusion weighted imaging (DWI) in all cases. Other imaging sequences included susceptibility-weighted imaging (SWI) in 21 patients, axial gradient recalled-echo (GRE) T2*WI in 4 patients, both SWI and GRE T2*WI in 3 patients, time-of-flight MR angiography (TOF-MRA) in 18 patients, and two-dimensional time-of-flight MR venography (2D-TOF-MRV) in 17 patients. Scanning parameters are summarized in the supplementary table.

### Image Analysis

A board-certified neuroradiologist with a certificate of added qualification in pediatric neuroradiology (A.S.), blinded to clinical information and outcomes, conducted the image analysis. Clinical images were retrospectively reviewed on the clinical PACS. A standardized data-capture form was conducted for the following: the location, size, signal intensity of the subpial hemorrhage, the underlying brain parenchyma changes, distribution of concomitant hemorrhages combined with subpial hemorrhage, as well as the presence of the yin-yang sign, the hyperintense pia mater sign (HPm-sign), and prominent perimedullary veins (PMV).

The size of the subpial hemorrhages was quantified in three dimensions: "x," perpendicular to the cortex, and "y" and "z," parallel to the cortex and perpendicular to each other. These dimensions were measured at the largest diameter. A cutoff of 5 mm for the "x" dimension and 10 mm for the "y" and "z" dimensions was used to classify the hemorrhages into the following categories: "very small" (all three dimensions below the cutoff), "small" (one dimension above the cutoff), "medium" (two dimensions above the cutoff), and "large" (all three dimensions above the cutoff), as previously described by Dabrowski et al. [2]. When there were multifocal subpial hemorrhages, the largest lesion was measured.

Based on the T2WI and T1WI appearance, three patterns of subpial hemorrhage were identified [9]. In pattern 1, the subpial collection demonstrated T1 hyperintense and T2 hypointense signal; in pattern 2, T1 and T2 hyperintense signal, and in pattern 3 the subpial collection demonstrated mixed T2 signal. The appearance of the underlying brain parenchyma was assessed on T1WI, T2WI, DWI and SWI/GRE T2*WI, on initial MRI. Two groups were identified; cortical infarct without hemorrhage, and cortical infarct with hemorrhage.

Follow-up MRI scans when available were analyzed to assess the natural course of the disease.

### Statistical analysis

Data were analyzed using descriptive statistics, including proportions, percentages, medians, and ranges for non-normally distributed variables, to summarize demographics, clinical information, and MRI findings. Statistical comparisons of groups-categorical data (presence/absence of intraparenchymal or intraventricular hematoma versus PMV) were conducted using Fisher´s exact test. Two-sided P values ≤ 0.05 were considered statistically significant. All analyses were performed using Excel 2016 (Microsoft) and SPSS Version 29 (SPSS, Chicago, USA).

## Results

### Demographics

Twenty-eight patients with subpial hemorrhage were included in this study between January 2010 and December 2023, 13 of whom were female and 15 male. The median age at disease onset was 12 h (range 1–336). Most (82%) were born at term gestation (≥ 37 weeks), 7% were moderate-to-late preterm (32–37 weeks), 4% were very preterm (28–32 weeks) and 7% was extremely preterm (< 28 weeks). The mean APGAR score at 1 min and 5 min was 9 (range 1–10) and 9 (range 4–10), respectively.

### Clinical presentation and associated medical or surgical conditions

The following pregnancy complications were observed in our cohort: gestational diabetes in four cases, polyhydramnios in two cases, pre-eclampsia in two cases, twin pregnancy in one case, and a history of controlled gestational hypertension in two patients.

The most common clinical presentation was a combination of apneic events and clinical seizures (50%). Associated conditions included hematologic abnormalities in 15 of 26 patients (58%), with elevated D-dimer, thrombocytopenia, and activated protein C being the most common causes. Additional associated conditions included: hypoxic-ischemic encephalopathy (HIE) in 3, hydrocephalus in 3, infection in 2, congenital heart disease in 2, birth trauma in 2, and cardiorespiratory failure in 1. In our cohort, two neurosurgical interventions were performed. One patient, a very preterm neonate, underwent ventriculosubgaleal shunt placement for hydrocephalus secondary to intraventricular haemorrhage (IVH). Additionally, another patient with a large subpial hemorrhage, associated with cortical infarction and intraparenchymal haemorrhage (IPH), required evacuation of the subpial hemorrhage due to significant mass effect.

A summary of patient demographics, imaging indications, clinical presentations, hematologic abnormalities, treatments and outcomes is provided in Table 1.

**Table 1:** Patient demographics, clinical data, and outcome

Sample characteristics	Subgroups	Result n (%)
Birth/mode of delivery		
Gestational age	Term (≥ 37)	23/28 (82%)
	Moderate-to-late pretem (32–37)	2/28 (7%)
	Very preterm (28–32)	1/28 (4%)
	Extremely pretem (≤ 28)	2/28 (7%)
Mode of delivery	Vaginal	14/19 (74%)
	Instrumental delivery	2/19 (11%)
	Cesarean delivery	3/19 (16%)
Apgar (median,range)	1 min	18/28 9 (1–10)
	5 min	18/28 9 (4–10)
Sex	Male	15/28 (54%)
	Female	13/28 (46%)
Age onset symptoms (hours median, range)		25/28 12 (1–336)
Age at initial MRI (days median, range)		28/28 3 (1–51)
Imaging indications/Clinical presentation	Apnea	4/28 (14%)
	Seizures	10/28 (36%)
	Apnea + seizures	14/28 (50%)
	Birth trauma	2/28 (7%)
	Postneurosurgery imaging	2 /28 (7%)
	Routine NICU screening	2/28 (7%)
	Cardiorespiratory failure	1/28 (4%)
	HIE	3/28 (11%)
	Infections*	2/28 (7%)
	Congenital heart disease	2/28 (7%)
	Hydrocephalus	3/28 (11%)
Hematological abnormalities		15/26 (58%)
	Elevated D-dimer	6/26 (23%)
	Thrombocytopenia	4/26 (15%)
	Activated Protein C	4/26 (15%)
	P-fibrinogen	2/26 (8%)
	Elevated PT/PTT	2/26 (8%)
	Factor VII deficiency	1/26 (4%)
	P-INR	3/26 (12%)
Age at more recent follow-up MRI (months median, range)		9/28 3 (2–60)
Outcome	No neurologic deficit	13/20 (65%)
	With neurologic deficit	5/20 (25%)
	Motor delay	2/20 (10%)
	Epilepsy	2/20 (10%)
	Motor delay + epilepsy	1/20 (5%)
	Died	2/20 (10%)

HIE hypoxic-ischemic encephalopathy, P-INR international normalized ratio, PT/PTT prothrombin time/partial thromboplastin time, * Group *B Streptococcus* meningitis.

### Neuroimaging characteristics

In this study, 24 patients (86%) had unilateral subpial hemorrhages, while 4 patients (14%) presented with multiple hemorrhages. Subpial hemorrhages were located in all areas of the cortex with a predilection for the temporal lobe (44%), followed by the frontal (31%), the parietal (22%), and occipital lobes (3%). No subpial hemorrhages were identified in the posterior fossa. Lesion laterality (left:right) was 56%:44%.

**Fig. 1:**
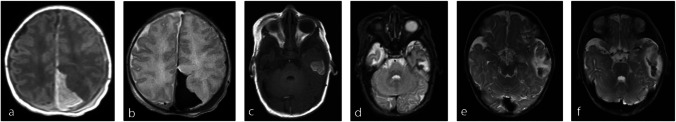
Patterns of neonatal subpial hemorrhage. Pattern 1. Brain MRI in a 2-day-old term male with apnea and seizures (**a-b**). Axial T1-weighted (**a**) and T2-weighted images (**b**) showing a hematoma with a high signal intensity and low signal intensity respectively along the medial side of the left parietal lobe. Pattern 2. Brain MRI in a 4-day-old term male with apnea, seizure and therapeutic hypothermia for HIE (**c-d**). Axial T1-weighted (**c**) and T2-weighted images (**d**) showing a hematoma with high signal intensity in the left temporal lobe. Pattern 3. Brain MRI in a 7-weeks-old term female with atrioventricular septal defect and pulmonary hypertension (**e–f**). Axial T2-weighted images (**e, f**) showing a hematoma with mixed signal intensity in the left temporal lobe

**Fig. 2:**
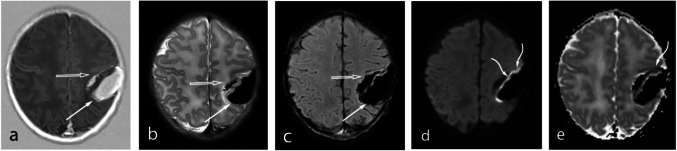
Neonatal subpial hemorrhage with underlying cortical infarct and small white matter hemorrhage. Brain MRI in a 5-day-old term male with seizures (**a-e**). Axial T1-weighted image (**a**) shows high signal intensity, T2-weighted (**b**) and susceptibility-weighted (**c**) images show low signal intensity of subpial hemorrhage on left parietal lobe overlying cortex and the precentral gyrus (arrows). Axial DWI (**d**) and ADC (**e**) maps show decreased diffusivity indicating underlying cortical infarct (curved arrows). Note small hemorrhage in the underlying white matter (open arrows in **a**,** b**,** c**)

**Fig. 3:**
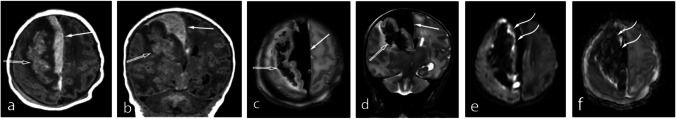
Neonatal subpial hemorrhage with underlying cortical infarct and large white matter hemorrhage. Brain MRI in a 2-day-old term female with forceps delivery, seizures (**a-f**). Axial and coronal T1-weighted (**a, b**) and T2-weighted (**c, d**) images show high and low signal intensity of a large subpial hemorrhage along the medial aspects of the right frontal/parietal lobes (arrows). Axial DWI (**e**) and ADC (**f**) maps shows extensive cytotoxic edema of the underlying cortices (curved arrows). Note large parenchymal hemorrhage in the underlying white matter (open arrows in **a, b, c, d**)

Subpial hemorrhages in twenty-three patients (82%) were categorized as large, in four (14%) as medium, in one (4%) as small. No patients were recorded with very small hemorrhages. Imaging analysis identified three distinct patterns of subpial hemorrhage on T1WI and T2WI. Pattern 1, the most common, was observed in 89% of cases and is characteristic of a typical acute or early subacute hemorrhage; in these cases, the MRI was performed at an age of 3 days (range, 1–15 days of life) (Fig. 1). The following findings of the underlying brain parenchyma were found: parenchymal cytotoxic edema was present subjacent to the subpial hemorrhages in all cases, and IPH in 86% of patients (Fig. 2, 3). Prominence of the medullary veins was noted in 82% of cases.

**Fig. 4:**
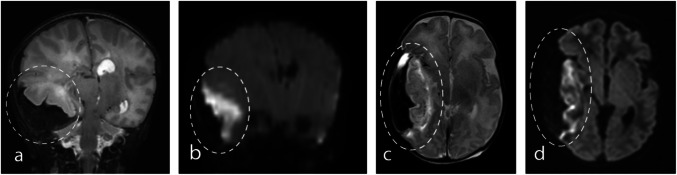
Neonatal subpial hemorrhages with Yin-yang sign. Brain MRI in a 1-day-old term male with apneic events and seizures (**a-b**). Coronal T2-weighted image (**a**) shows low signal intensity corresponding to subpial hemorrhage splaying underlying sulcus, and DWI (**b**) shows a bright underlying cerebral cortex with cytotoxic edema of temporal lobe cortical ribbon indicating underlying infarcted brain parenchyma (dotted circles). Brain MRI in a 6-day-old term male with forceps delivery and apnea (**c-d**). Axial T2-weighted image (**c**) and DWI (**d**) show the combination of a dark subpial hemorrhage and a bright underlying cerebral cortex (extensive cytotoxic edema) resembling the yin-yang symbol ( dotted circles)

**Fig. 5:**
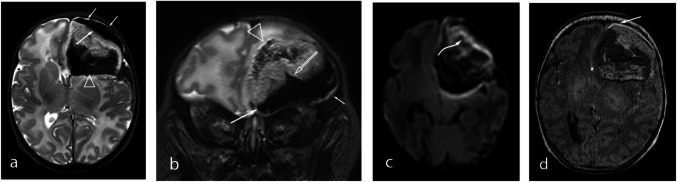
Neonatal subpial hemorrhage with the hyperintense pia mater sign. Brain MRI in a 2-old-day term female with apneic events and seizures (**a-d**). Axial and coronal T2-weighted images (**a, b**) show a low signal intensity subpial hemorrhage (arrows) splaying underlying sulcus (open arrow) on left frontal lobe separated from CSF (short arrows) with associated subcortical white matter hemorrhage (open arrowheads). Note mass effect. Axial DWI (**c**) shows cytotoxic edema of right frontal lobe cortical ribbon (curved arrow) due to acute cortical infarction. Axial TOF-MRA (**d**) demonstrates a linear high signal intensity surrounding the outer contour of subpial hemorrhage, distinctly separated from both cortical ribbon and the subpial hemorrhage indicative of a blood-stained lifted-off pia matter (arrow)

**Fig. 6:**
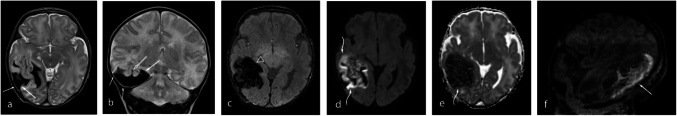
Neonatal subpial hemorrhage with the hyperintense pia mater sign. Brain MRI in a 3-old-day term male with apnea and seizures (**a-f**). Axial and coronal T2-weighted images (**a**, **b**) show a low signal intensity subpial hemorrhage (arrows) splaying underlying sulcus (open arrow) on right temporal lobe separated from the CSF (short arrows). SWI (**c**) shows a severe hemorrhage in the underlying white mater (open arrowhead). Axial DWI (**d**) and ADC (**e**) maps demonstrate cortical infarct (curved arrows). Axial TOF-MRI (**f**) demonstrates the HPm-sign (arrow)

In this study, the yin-yang sign was identified in twenty-one patients. This sign is characterized by a combination of a dark, hypointense subpial hemorrhage paired with an underlying bright, hyperintense cerebral cortex indicating infarcted parenchyma on T2WI/DWI (Fig. 4). Among the 18 patients who underwent TOF-MRA, a linear hyperintensity surrounding the external contour of the subpial hemorrhage was identified in 12 cases (Fig. 5 and 6). Of the 17 patients who underwent MRV, one was found to have a persistent falcine sinus, which is an anatomic variant. However, all MRV examinations showed no evidence of thrombosis in the dural venous sinus or cerebral veins.

**Fig. 7:**
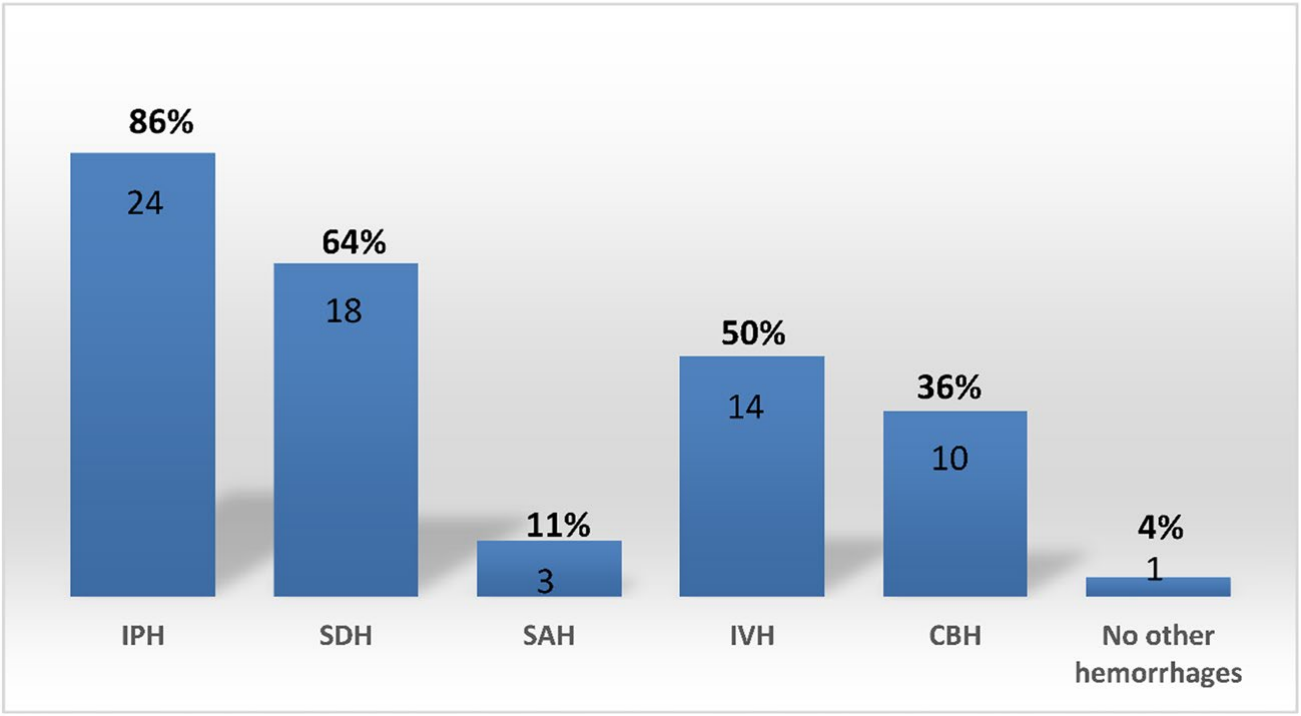
Distribution of concomitant hemorrhages in neonates with subpial hemorhage by location. IPH indicates intraparenchymal hemorrhage; SDH, subdural hematoma; SAH, subarachnoid hemorrhage; IVH, intraventricular hemorrhage; CBH, cerebellar hemorrhage

**Fig. 8:**
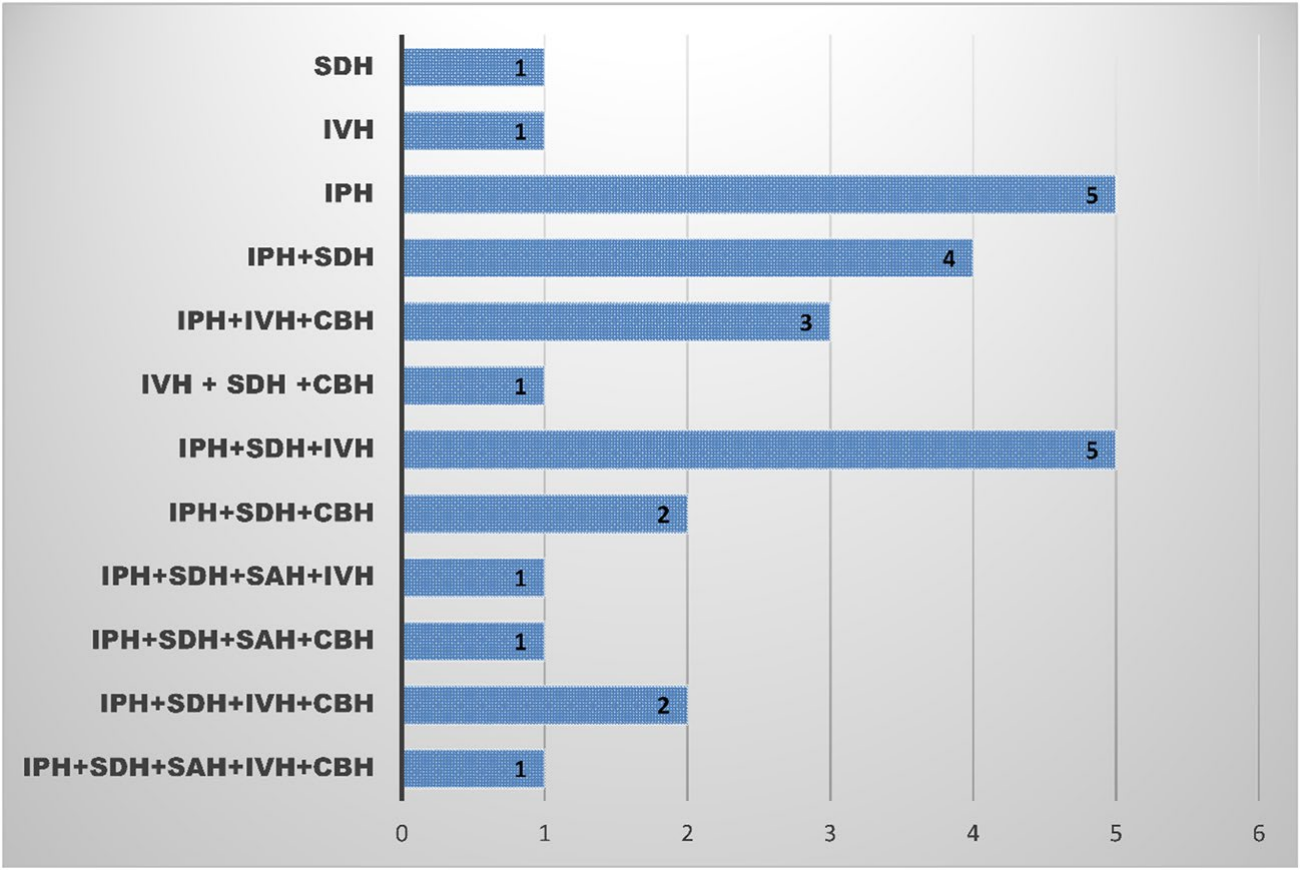
Distribution of overlapping hemorrhages neonates with subpial hemorrhage. IPH indicates intraparenchymal hemorrhage; SDH, subdural hematoma; SAH, subarachnoid hemorrhage; IVH, intraventricular hemorrhage; CBH, cerebellar hemorrhage

Subpial hemorrhage was found to be associated with at least one other type of intracranial hemorrhage (ICH) in 27 patients (96%). The specific types of associated hemorrhages included: IPH in 24 patients (86%), subdural hematoma (SDH) in 18 patients (64%), IVH in 14 patients (50%), cerebellar hemorrhage (CBH) in 10 patients (36%), subarachnoid hemorrhage (SAH) in 3 patients (11%) (Fig. 7). Additionally, multiple overlapping types of ICHs were present in 71% of cases (Fig. 8). There was a strong correlation between PMV and IPH (p = 0,028), with 87% of infants with IPH showing an association with PMV; in contrast, no statistical association was found between IVH and PMV (p = 0.326), as only 56,5% of infants with IVH had PMV. The results of the initial MRI are summarized in Table 2.

**Table 2:** MRI findings in neonates with subpial hemorrhage at presentation

MRI findings		N (%)
Location	Unilateral	24/28 (86%)
	Multifocal	4/28 (14%)
	Temporal	14/32 (44%)
	Frontal	10/32 (31%)
	Parietal	7 /32 (22%)
	Occipital	1/32 (3%)
Lesion laterality	Left	18/32 (56%)
	Right	14/32 (44%)
Subpial hemorrhage	Pattern 1	25/28 (89%)
	Pattern 2	1/28 (4%)
	Pattern 3	2/28 (7%)
Size subpial hemorrhage	Very small	0/28 (0%)
	Small	1/28 (4%)
	Medium	4/28 (14%)
	Large	23/28 (82%)
Adjacent brain parenchyma	Parenchymal cytotoxic edema	28/28 (100%)
	IPH	24/28 (86%)
	Cortical infarct with no IPH	4/28 (14%)
	Cortical infarct with IPH	24/28 (86%)
Yin-yang sign		21/28 (75%)
Midline shift		15/28 (54%)
Hyperintense pia mater sign		12/18 (67%)
Prominent medullary veins		23/28 (82%)
Concomitant intracranial hemorrhages		27/28 (96%)
	IPH	24/28 (86%)
	SDH	18/28 (64%)
	SAH	3/28 (11%)
	IVH	14/28 (50%)
	CBH	10/28 (36%)
	Multiple compartments	20/28 (71%)
	NOH	1/28 (4%)
Associated conditions	Acute ischemic stroke	1/28 (4%)
	HIE	3/28 (11%)
	Anatomic anomalies	1/28 (4%)
	Hydrocephalus	3/28 (11%)

CBH cerebellar hemorrhage, HIE hypoxic-ischemic encephalopathy, IPH intraparenchymal.

hemorrhage, IVH intraventricular hemorrhage, NOH no other hemorrhage, SAH subarachnoid.

hemorrhage, SDH subdural hemorrhage.

### Follow-up MR Imaging

Among the 28 patients, 9 underwent follow-up MRI, with a median follow-up period of 3 months (range 2 to 60 months). The follow-up scans varied as follows: 4 patients had 1 scan, 2 had 2 scans, 1 had 3 scans, 1 had 4 scans, and 1 had 14 scans. In six of these patients, with a median follow-up period of 13,5 months (range, 2–60 months), the subpial hemorrhage evolved into a fluid collection with no mass effect and a reduction in the size of the underlying cerebral infarct. In two patients, with follow-up MRI intervals of 2 and 4 months, respectively, the subpial hemorrhage evolved into fluid collections with reduced mass effect and also showed a decrease in the size of the underlying cerebral infarct. In one patient, with a follow-up interval of 3 months, both the subpial hemorrhage and the underlying cerebral infarct remained stable, maintaining similar size and shape.

### Outcomes

Clinical follow-up data were available for 20 out of the 28 patients. Among these, two patients died: the first, an extremely preterm infant due to factors related to cardiorespiratory failure, while the second, a moderate-to-late preterm infant due to the combination of HIE, congenital diaphragmatic hernia and pulmonary hypertension.

At follow-up, 5 patients (25%) presented with neurologic deficits, including 2 patients with motor delay, 2 patients developed remote symptomatic epilepsy, and 1 patient with both motor delayed and epilepsy.

## Discussion

Neonatal ICHs include various types, such as IPH, IVH, SAH, SDH, and subpial hemorrhage. This retrospective study focuses on subpial hemorrhage, a subtype of ICH that has been underreported and not well understood until recently [1, 2, 8]. We investigate the demographic, pathophysiologic, clinical, and neuroimaging characteristics of subpial hemorrhage, emphasizing its distinct neuroimaging features that differentiate it from other extraaxial hemorrhages. Subpial hemorrhage may be associated with poor neurological outcomes, likely due to the extent of parenchymal injury [1]. Our results demonstrate a correlation between PMV and IPH, and introduce the HPm-sign as a promising indicator for differentiating subpial hemorrhage from other neonatal hemorrhage types in certain cases.

Our cohort of 28 patients with subpial hemorrhage included 5 preterm infants (two moderate-to-late preterm, one very preterm, and two extremely preterm). Of these, one survived without neurological sequelae, one developed quadriplegic cerebral palsy and epilepsy, and two died; follow-up was unavailable for one patient. These results are quite similar to those of Assis et al. [8], who reported a 60% mortality rate among premature infants, while our study found a 40% mortality rate. In contrast, Pinto et al. [9] did not identify worse outcomes for early preterm infants compared to others.

Hematologic abnormalities were found in 58% of our patients, which is lower than the rates reported by Cain et al. [7], but higher compared to Assis et al. [8], where only 1 of 16 patients had abnormal coagulation profiles. The most common abnormalities in our cohort were elevated D-dimer (23%), thrombocytopenia (15%), and increased activated protein C (15%). In contrast, Cain et al. [7] reported elevated D-dimer in 53% of patients, while another study found thrombocytopenia in 60% of neonates [9].

In our study, 86% of the lesions were unilateral, similar to the reported incidence by Zhuang et al. [10], but differing from Assis et al. [8], where all lesions were unilateral. Most lesions were located in the temporal lobes, consistent with other studies [8–10]. The occipital lobe had the lowest frequency of involvement, which agrees with previous studies [8, 9] but contrasts with Zhuang et al. [10], who reported it as the second most frequently affected region (29.4%). It has been postulated that the predominance of temporal lobe subpial hemorrhages may be related to skull deformation during birth at the confluence of sutures at the pterion, while occipital hemorrhages may be associated with the asterion, corresponding to the venous drainage territories of the superficial middle cerebral vein and the vein of Labbé [6, 11].

All patients in our study with subpial hemorrhage had underlying cortical infarcts, which is consistent with findings from other authors [7–9]. However, Zhuang et al. [10] reported 6 of 34 patients presenting with subpial hemorrhage only. Additionally, we found concurrent IPH in 86% of cases, similar to the 82% reported by Cain et al. [7], but higher than in other studies [8, 10] and lower than in Pinto et al. [9].

In 21 patients (75%), the distinct imaging pattern resembling the yin-yang symbol was observed, with a T2WI hypointense component corresponding to the subpial hemorrhage extending to the cerebral sulci and a T2WI hyperintense component reflecting the underlying infarcted cerebral cortex. Previous studies have reported the yin-yang sign in 12 of 34 patients [10], 9 of 10 patients [9] and all patients [8].

An interesting finding in our study was the visualization of a hyperintense line surrounding the external contour of the subpial hemorrhage on TOF-MRA in 12 of 18 patients, which we propose to call the hyperintense pia mater sign (HPm-sign). This is the first systematic report highlighting the value of TOF-MRA in diagnosing this condition; Barreto et al. [1] described only one case, suggesting that this finding indicates blood-stained pia mater. Similar to Barreto et al. [1], we believe that the HPm-sign could assist neuroradiologists in differentiating this type of hemorrhage from other intracranial hemorrhages in neonates in certain cases.

In our study, subpial hemorrhage was associated with another type of ICH in 96% of cases, most commonly parenchymal (86%) and subdural (64%) hemorrhages. Compared to previous studies, our incidence of IPH was higher (73%, 77%, and 64%) [2, 8, 10], but similar to that reported by Pinto et al. [9].

CBHs were identified in 36% of our patients, a significant finding considering that it has only been reported in one previous study, which identified bilateral cerebellar microbleeds in 60% of neonates [9]. Infants with CBH often have significantly lower gestational ages and higher rates of intubation at birth, hypotension, IVH, and sepsis [12]. In our cohort, all 5 premature patients had CBH, and 6 of 13 patients with IVH also presented with CBH. Furthermore, 4 of 10 patients with CBH required intubation, compared to 7 of 18 without cerebellar involvement.

It has been proposed that medullary venous congestion may play a critical role in the pathophysiology of subpial hemorrhage [2, 6–8]. In our study, prominence of the medullary veins, indicating congestion and/or thrombosis, was observed in 82%, consistent with findings from other authors [7, 9]. We also noted frequent occurrences of parenchymal and intraventricular hemorrhages, both of which are common in neonates with venous thrombosis [7, 13, 14]. Our findings indicate a significant correlation between PMV and IPH, supporting the theory that medullary vein congestion is central to the pathophysiology of subpial hemorrhage.

The subpial space is defined as the potential space bordered externally by the pia mater and internally by the external glial limiting membrane (glia limitans) [1, 3, 4, 15, 16]. Pial arteries run parallel to the cerebral cortex surface, encased by a single layer of pia mater that extends as a leptomeningeal sheath into the brain, separated from the glia limitans on the surface of the cortex by the subpial space. Veins in the subpial space may or may not be surrounded by pia [4, 16, 17], potentially increasing their rupture risk in this space [1–3]. Subsequent blood accumulation in the subpial space can compress the underlying cerebral parenchyma, leading to venous congestion, medullary vein thrombosis, and cortical infarction [1, 8]. Marín-Padilla in a neuropathological study found that subpial hemorrhages are invariable associated with focal disruptions of the external glial limiting membrane, observing disintegration of glial endfeet, rupture of perforating vessels, subpial hemorrhages, damaged capillaries with rupture of their wall and focal thrombosis in acute lesions [3].

On follow-up MR images, neither the subpial hemorrhage nor the underlying infarct progressed over time, consistent with findings by Assis et al. [8]. The average age at the most recent clinical follow-up was 15 months, ranging from 1 to 144 months. Two of 20 patients died (10%), but none due to subpial hemorrhage. Other studies reported mortality rates of 6% [7], 10% [9], and 19% [8]. In our study, 25% of surviving patients had neurological deficits, compared to 44% and 12.5% in two other studies [7, 8]. In our cohort, 10% exhibited motor delays, which is lower than the rates reported by Dabrowski et al. [2] and Pinto et al. [9], where 76% and 50% had motor delays, respectively. Only two patients (10%) developed remote symptomatic epilepsy, similar to previous studies that found rates of 6% and 10% [7, 9], but lower than Dabrowski et al. [2], who reported 24%.

This study has several limitations that should be considered, primarily related to its small sample size and retrospective design. Another limitation of our study is that outcomes are likely confounded by comorbid conditions and other concurrent neurological insults.

## Conclusion

Subpial hemorrhage is a rare and distinctive subtype of ICH in neonates, characterized by unique imaging features. These include two primary components: the subpial hemorrhage and signal changes in the underlying brain parenchyma, such as cortical infarcts and, in most cases, white matter hemorrhage. This study is the largest to systematically identify the HPm-sign on TOF-MRA, which may assist neuroradiologists in distinguishing subpial hemorrhage from other types of neonatal hemorrhages in some cases. We found a correlation between PMV and IPH, supporting the theory that medullary vein congestion plays a key role in the pathophysiology of subpial hemorrhage. Further research is essential to better understand the underlying mechanisms and to assess the long-term neurological outcomes in neonates affected by subpial hemorrhage.

## Supplementary Information

Below is the link to the electronic supplementary material.

Supplementary file1 (DOCX 13 KB)

## Data Availability

The datasets generated during and/or analysed during the current study are available from the corresponding author on reasonable request.

## Abbreviations

CBH: Cerebellar hemorrhage
DWI: Diffusion weighted-imaging
GRE: Gradient recalled-echo
HPm-sign: Hyperintense pia mater sign
IPH: Intraparenchymal hemorrhage
IVH: Intraventricular hemorrhage
MRI: Magnetic Resonance Imaging
NOH: No other hemorrhage
P-INR: International normalized ratio
PMV: Prominent medullary veins
PT: Prothrombin time
PTT: Partial thromboplastin time
SAH: Subarachnoid hemorrhage
SDH: Subdural hematoma
SWI: Susceptibility–weighted imaging
TOF MRA: Time-of-flight MR angiography
TOF-MRV: Time-of-flight MR venography
