# Supplementary material for: Neonatal subpial hemorrhage: clinical presentation, neuroimaging findings and outcome
Source: Neuroradiology. 2025 Mar 17;67(4):1071–80. doi: 10.1007/s00234-025-03589-y (PMC12041188; doi:10.1007/s00234-025-03589-y)
Supplement: Supplementary file 1 — Supplementary file1 (DOCX 13 KB) [file 234_2025_3589_MOESM1_ESM.docx]

**Supplementary Table**.

| **Sequences** | **3T MR** | | | | **1,5T** | | | |
| --- | --- | --- | --- | --- | --- | --- | --- | --- |
|  | TR | TE | Thickness | Gap | TR | TE | Thickness | Gap |
| **T1 3D-MPRAGE** | 2200 | 2,98 | 1 | 0 | 2000 | 2,28 | 0,9 | 0 |
| **T1IR** | 2500 | 13 | 2,5 | 0 | 7000 | 2,28 | 0,9 | 0 |
| **T2** | 6000 | 143 | 2,5 | 0 | 6000 | 147 | 4 | 1,2 mm 30% |
| **GRE T2*** |  |  |  |  | 800 | 147 | 4 | 1,2 mm 30% |
| **SWI** | 30 | 20 | 1,4 | 0 | 39 | 30 | 3 | 0 |
| **DWI** | 5430 | TE1 68 TE2 113 | 3 | 0,6 mm 20% | 3970 | TE1 68 TE2 114 | 4 | 1,2 mm 30% |
| **MRA 3D-TOF** | 22 | 3,58 | 0,5 | 0,4 mm 20% | 24 | 7 | 0,5 | 0,8 mm 30% |
| **MRV** | 67,1 | 6,84 | 0,8 | 0 | 57,05 | 9,93 | 1 | 0 |
